# Stereotactic arrhythmia radioablation and its implications for modern cardiac electrophysiology: results of an EHRA survey

**DOI:** 10.1093/europace/euae110

**Published:** 2024-04-26

**Authors:** Boldizsar Kovacs, Helge Immo Lehmann, Martin Manninger, Ardan Muammer Saguner, Piotr Futyma, David Duncker, Julian Chun

**Affiliations:** Department of Cardiology, University of Michigan, 1500 East Medical Center Drive, Ann Arbor, 48109 MI, USA; Department of Cardiology, University Heart Center Zurich, Raemistrasse 100, Zurich 8091, Switzerland; Department of Cardiology, University of Michigan, 1500 East Medical Center Drive, Ann Arbor, 48109 MI, USA; Corrigan Minehan Heart Center, Massachusetts General Hospital, 55 Fruit St, Boston, 02114 MA, USA; Division of Cardiology, Department of Medicine, Medical University of Graz, Graz, Austria; Department of Cardiology, University Heart Center Zurich, Raemistrasse 100, Zurich 8091, Switzerland; Medical College, University of Rzeszów and St. Joseph’s Heart Rhythm Center, Rzeszów, Poland; Hannover Heart Rhythm Center, Department of Cardiology and Angiology, Hannover Medical School, Hannover, Germany; Cardioangiologisches Centrum Bethanien, Agaplesion Bethanien Krankenhaus, Frankfurt, Germany

**Keywords:** Stereotactic arrhythmia radioablation, Survey, European Heart Rhythm Association

## Abstract

Stereotactic arrhythmia radioablation (STAR) is a treatment option for recurrent ventricular tachycardia/fibrillation (VT/VF) in patients with structural heart disease (SHD). The current and future role of STAR as viewed by cardiologists is unknown. The study aimed to assess the current role, barriers to application, and expected future role of STAR. An online survey consisting of 20 questions on baseline demographics, awareness/access, current use, and the future role of STAR was conducted. A total of 129 international participants completed the survey [mean age 43 ± 11 years, 25 (16.4%) female]. Ninety-one (59.9%) participants were electrophysiologists. Nine participants (7%) were unaware of STAR as a therapeutic option. Sixty-four (49.6%) had access to STAR, while 62 (48.1%) had treated/referred a patient for treatment. Common primary indications for STAR were recurrent VT/VF in SHD (45%), recurrent VT/VF without SHD (7.8%), or premature ventricular contraction (3.9%). Reported main advantages of STAR were efficacy in the treatment of arrhythmias not amenable to conventional treatment (49%) and non-invasive treatment approach with overall low expected acute and short-term procedural risk (23%). Most respondents have foreseen a future clinical role of STAR in the treatment of VT/VF with or without underlying SHD (72% and 75%, respectively), although only a minority expected a first-line indication for it (7% and 5%, respectively). Stereotactic arrhythmia radioablation as a novel treatment option of recurrent VT appears to gain acceptance within the cardiology community. Further trials are critical to further define efficacy, patient populations, as well as the appropriate clinical use for the treatment of VT.

## Graphical Abstract

**Graphical Abstract:**
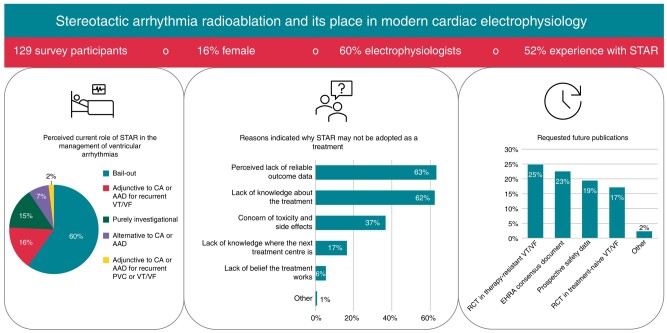

**What’s new?**

- Stereotactic arrhythmia radioablation (STAR) is an emerging treatment option in electrophysiology; however, the current acceptance and future role of STAR among cardiologists is unknown.
- Reported main advantages were efficacy in the treatment of arrhythmias not amenable to conventional treatment and a non-invasive treatment approach with overall low expected acute and short-term procedural risk.
- In this survey, we show that STAR is gaining acceptance within the cardiology and subspecialized electrophysiology community; further data from randomized trials appear critical to define best patient populations and the appropriate clinical use.

## Introduction

Despite technological and clinical advancement in the field of cardiac electrophysiology, ablation of ventricular tachycardia (VT) remains challenging. Recurrences often occur due to deep myocardial substrates that cannot be reached using radiofrequency energy for ablation.^1–4^ The use of x-ray beams for myocardial ablation has been proposed as a means to overcome the challenges of catheter ablation (CA) and also created enthusiasm for a fully non-invasive ablation approach.^5–9^ Subsequently, the first published cases where stereotactic arrhythmia radioablation (STAR) was used for ablation of ventricular tachycardia (VT) were performed in 2013 and 2014.^10,11^ The aim of STAR therefore is comparable with that of a CA procedure, where the goal is the creation of transmural fibrosis in order to abolish the arrhythmogenic substrate. Interestingly, the commonly prescribed radiation doses are insufficient to cause transmural fibrosis and alternative antiarrhythmic mechanisms have been performed and are being investigated^7–9,12^ (NCT 06299176).

Over the past decade, STAR has gained attention within the electrophysiology community in particular for the treatment of recurrent VT after multiple attempts of CA.^13,14^ To date, numerous case reports and small cohort studies, but no randomized controlled trials (RCTs), have been published in this arena.^15–22^ Thus, there is lack of any recommendations in the most recent European Society of Cardiology VT/ventricular fibrillation (VF) guidelines regarding the clinical application of STAR.^23^ Given the gap in clinical data, but also as STAR is a new treatment in a highly collaborative field between radiation oncology, radiology and cardiac electrophysiology, it is currently unknown what the acceptance and use of STAR is in clinical practice and what the perceived future role of this therapeutic tool is in practice. The goal of this European Heart Rhythm Association (EHRA) survey was to assess the current role of STAR within the cardiology community, barriers to its use, and the expected future role in clinical care of arrhythmia patients.

## Methods

An anonymous online survey was performed between 6 June and 5 July 2023. The questionnaire was prepared on SurveyMonkey by the authors with the support of the Scientific Initiatives Committee of the EHRA. Distribution of the questionnaire was done via the EHRA newsletter, and on social media as well as the personal mailing lists among cardiologists. All cardiologists irrespective of EHRA membership, geographical location of practice, or cardiology subspecialty were invited to participate. The survey consisted of 20 multiple-choice questions with focus on baseline demographics, awareness and access to STAR, current use of STAR, and future role of STAR based on prior clinical experience and scientific uncertainties expressed in prior publications (Supplementary material).^24–26^

### Statistical analysis

Continuous variables are presented as mean (±standard deviation). Comparisons were performed using χ^2^ or Fisher’s exact test, as appropriate. Questionnaires with relevant missing data were excluded case wise. All statistical analyses were performed with SPSS v29 (IBM Corp.), and figures were created using Excel (Microsoft).

## Results

### Baseline characteristics

A total of 129 completed surveys were received. The mean age of the survey participants was 43 (±11) years, and 25 (16.4%) were female. The top five countries with the most participants were Germany (*n* = 17), Switzerland (*n* = 16), Poland (*n* = 10), USA (*n* = 10), and the Netherlands (*n* = 8) (supplementary materials). The most common primary specialty reported was cardiac electrophysiology (*n* = 91, 70.5%), followed by general cardiology (*n* = 13, 10%), cardiac imaging (*n* = 5, 3.8%), interventional cardiology (*n* = 4, 3.1%), heart failure (*n* = 3, 2.3%), or other (*n* = 3, 2.3%). Eighteen participants (13.9%) were fellows in training at the time of completing the survey. The mean time in cardiology practice reported was 10.6 (±10.2) years. The reported workplace was university hospital (*n* = 93, 72.1%), specialized public hospital (*n* = 18, 14%), private hospital (*n* = 10, 7.8%), district/community hospital (*n* = 4, 3.1%), private practice (*n* = 1, 0.8%), and other (*n* = 3, 2.3%).

### Awareness and access

Fifty-five participants (42.6%) had access to STAR in their institution for the clinical application, while it was available to 44 participants for both clinical and research purposes (34.1%). Eleven participants reported availability for research purposes only (8.5%). Sixty-five participants (50.4%) were aware of STAR as a therapeutic option but had no access to it in their institution. These study participants were evenly distributed among the participating countries (*P* = 0.37). The remaining nine participants (7%) were not aware of STAR as a therapeutic entity.

Almost half of the participants had no history of treating or referring a patient for STAR (*n* = 62, 48.1%). The median number of patients treated with or referred for STAR by the remaining participants was 4 (range 1–100). The most common indications for STAR were recurrent VT/VF in patients with structural heart disease (SHD, *n* = 58, 45%), recurrent VT/VF without the presence of SHD (*n* = 10, 7.8%), and PVC (*n* = 5, 3.9%). Forty-six participants (35.7%) followed patients after STAR had been performed.

### Current treatment and referral practices

**Figure 1:**
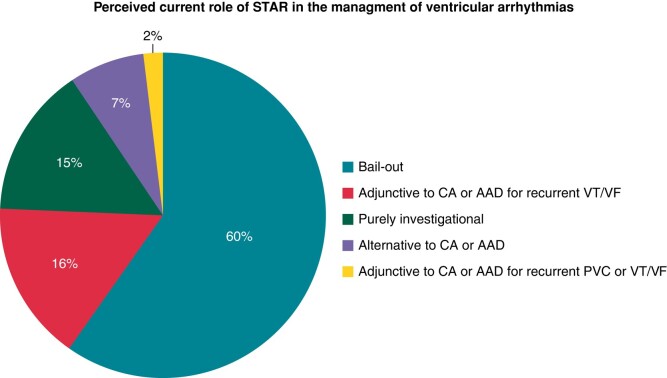
The perceived current role of STAR in the management of ventricular arrhythmias. STAR, stereotactic arrhythmia radioablation; CA, catheter ablation; AAD, antiarrhythmic drug; VT/VF, ventricular tachycardia/fibrillation; PVC, premature ventricular contraction.

The perceived current role of STAR is shown in *Figure 1*. The majority (115, 89%) only saw a role for STAR either as a bail-out, adjunctive to other established therapies, or as an investigational tool. Only 7% of the participants indicated that STAR may be an alternative to CA or antiarrhythmic drug therapy (AAD).

**Figure 2:**
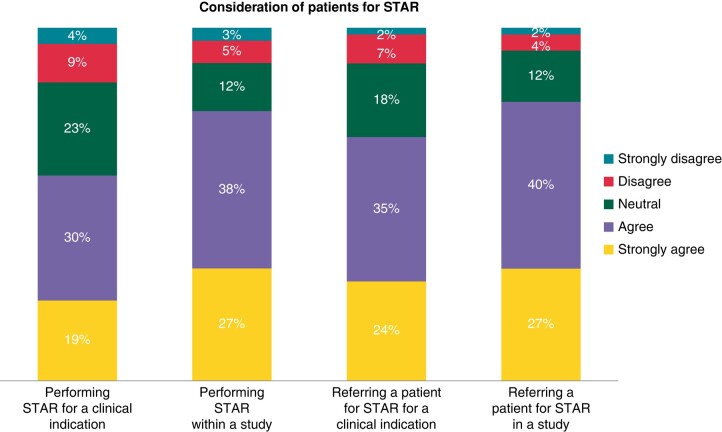
Consideration of patients for STAR. Survey participants indicated willingness to perform STAR or refer patients for STAR either in a clinical or research setting. STAR, stereotactic arrhythmia radioablation.

Close to half of the participants agreed that they would consider performing STAR on a patient or referring a patient for STAR for a clinical indication (49.6% and 58.9%, respectively). The willingness to perform STAR in a research setting or refer patients to a study reached higher acceptance among participants (65.1% and 67.4%, respectively). On the other hand, 13.2% participants would disagree with performing STAR for a clinical indication, and 8.6% disagreed with referring patients for STAR (*Figure 2*).

**Figure 3:**
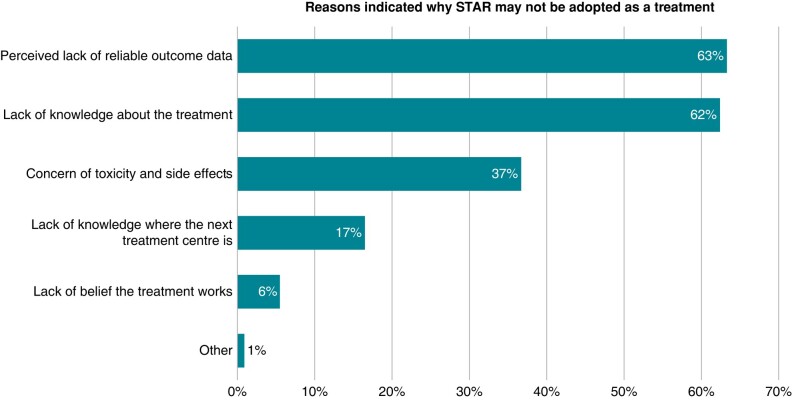
Reasons indicated why STAR may not be adopted as a treatment by surveyed physicians.

The reasons indicated against performing STAR are shown in *Figure 3*. The majority of participants perceived a lack of reliable outcomes data after STAR or indicated a lack of knowledge about the treatment. Importantly, only a minority (5.5%) believed that STAR did not work.

The survey participants ranked the following as the most important advantages of STAR: its efficacy in the treatment of arrhythmias not amenable to either CA or AADs (49%), a non-invasive treatment approach with overall low expected acute and short-term procedural risks (23%), and possible higher efficacy than other available treatment options (23%). Short procedural duration time, short recovery time from STAR treatment delivery, and reduction of AAD dose were perceived as the most important advantage by only 3%, 1%, and 1% of participants, respectively.

### Patient and institutional prerequisites

When considering patient referral for STAR, the most common patient characteristics required by the survey participants in order to consider STAR in a patient were one or more previous CA or contraindication for CA (79%), diagnosis of ischaemic or non-ischaemic cardiomyopathy (78%), recurrent monomorphic VT with >3 episodes within the preceding 3 months (68%), optimal antiarrhythmic medication (61%), or electrical storm (59%). The presence of an ICD was a requirement for only 39% of survey participants. In addition, pre-procedural investigations required prior to STAR were cross-sectional imaging (cardiac computed tomography or magnetic resonance imaging, 54.3%), 3D electroanatomic map (53.3%), non-invasive body mapping (20.9%), and 12-lead electrocardiogram (ECG) of the index arrhythmia (19.4%). A minority of participants (11.6%) indicated a lack of knowledge regarding the requisite pre-procedural investigations preceding STAR.

Survey participants regarded the following characteristics as a contraindication for STAR: pregnancy or breastfeeding (87%), life expectancy < 6 months (70%), temporary or genetic cause for VT (59%), eligibility for CA (58%), NYHA class IV heart failure (30%), polymorphic VT/VF (29%), ICD malfunction (24%), and prior chest irradiation (28%).

From an institutional perspective, VT ablation expertise was regarded as important when considering which anatomic locations should be irradiated (78%). On the other hand, radiation oncology expertise with STAR specifically was frequently not perceived as essential (only in 50%). In fact, 36% indicated expertise in any form of stereotactic body radiation therapy was required by an institution to perform STAR.

### Future directions

**Figure 4:**
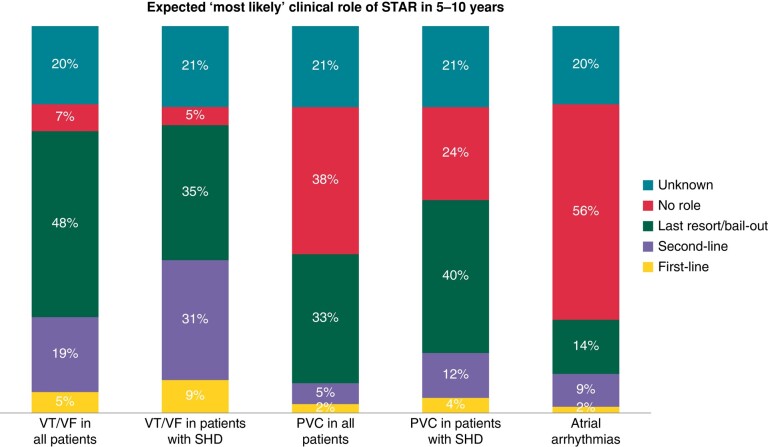
Expected most likely clinical role of STAR in 5–10 years. STAR, stereotactic arrhythmia radioablation; VT/VF, ventricular tachycardia/fibrillation; PVC, premature ventricular contraction; SHD, structural heart disease.

The majority of survey participants expected a clinical role for STAR in the treatment of VT/VF with or without underlying SHD in the majority of cases (72% and 75%, respectively), although only a minority expected a first-line indication for it (7% and 5%, respectively) and most viewed it as a bail-out option in the future (48% and 35%, respectively). In the context of treating PVCs and atrial arrhythmias, a notably smaller percentage of participants anticipate a prospective application of STAR in the future (*Figure 4*).

Survey participants were tasked with ranking the publication formats they deemed most critical for enhancing their clinical decision-making. The outcomes revealed that the most sought-after publication formats, as identified by the respondents, were RCTs in patients with recurrent VT/VF after CA ± AAD comparing STAR to repeat CA or AAD escalation (first place, 32%), European Society of Cardiology (ESC)/EHRA consensus documents (second place, 29%), prospective long-term safety data with documented evidence (third place, 25%), and RCT in treatment naive patients comparing STAR with CA or AAD (fourth place, 22%).

## Discussion

This is the first survey conducted on the current and future clinical role of STAR as perceived within the cardiology community.

### Main findings

- There is a widespread awareness of STAR (93%) as an antiarrhythmic therapeutic tool. Half of the survey participants work in an institution with access to STAR, and 60% have treated patients with (or referred patients for) STAR.
- The majority viewed both the current and future role of STAR as bail-out in instances of failed CA and ADD therapy for recurrent VT/VF (58%), regardless of underlying SHD, and only a minority considered it an option for the treatment of PVC (2%).
- The most common reported contraindications for STAR were pregnancy or breast-breastfeeding and life expectancy of <6 months. Interestingly, only 59% of respondents viewed temporary or genetic causes for VT as a contraindication for STAR.
- For the ideal clinical setting to perform STAR, expertise in VT ablation was deemed important, while expertise in radiation oncology specifically for STAR was considered secondary in importance.

### Awareness, familiarity, and access

There exists a considerable awareness of STAR, and already 60% of the survey participants administered STAR, with a median of four treated patients per participant. The treatment indication was recurrent VT/VF either with or without SHD, which is in line with available literature.^15–18,27–30^ The perceived role of STAR in these patients commonly aligns with a ‘bail-out’ strategy, serving as a last-resort intervention or as an adjunct following failed CA/AAD therapy, or purely investigational. Accordingly, only 7% of participants considered STAR as an alternative option and not only a bail-out.

In contrast, the treatment of PVCs with STAR was only deemed indicated and performed by a minority of participants similar to the available literature.^15,31,32^

As expected, the most important perceived advantage of STAR was in the treatment of recurrent treatment resistant arrhythmias with inaccessible substrate such as intramural VT/VF.^33^ This is an important message as radiation beams do not have limited tissue penetration such as radiofrequency or cryoenergies. Of note, the non-invasive nature with low expected complication rates was viewed as the most important advantage by 23% of the participants. This is an important consideration as CA for VT/VF is frequently performed in patients with advanced heart failure with a significant periprocedural morbidity and mortality^34^ and the use of haemodynamic support for VT ablation is not trivial.^35^ Similarly, although a short procedural and recovery time and reduction of AAD dose was viewed as the main advantage of STAR in only a minority of cases, all these factors may prevent prolonged hospitalizations and other complications. Previously reported STAR treatment durations have been maximally 112 min which is significantly lower compared with a mean duration of ∼300 min that has been reported for CA of VT in structural heart disease.^1,27,36^

### Patient and institutional prerequisites

A major technical limitation of STAR today is the localization of the arrhythmic substrate within the radiation treatment planning software. This currently involves transfer of 3D mapping data into a CT scan and a particular radiation treatment planning software. Despite this, just over half of the participants required pre-procedural electroanatomical mapping and cross-sectional imaging for the radiation treatment planning; this process in fact remains an area of active research and development of new approaches and thus possibly reflects the presence of multiple different solutions for different arrhythmias. It has been shown previously that radiation target delineation is dependent on the platform used, experience of the treating staff, and several different software solutions have been developed to improve this process.^37,38^ Most of these however are dependent on prior electroanatomical mapping and imaging to develop a treatment plan by radiation oncologists with expertise in STAR.

Although all available studies emphasize the importance of a multidisciplinary approach and cardiologists are not trained in planning radiation targets, target doses, surprisingly only 50% of survey participants viewed expertise of local radiation oncologists in STAR delivery as a prerequisite for treatment of patients, whereas expertise in VT ablation was considered a prerequisite in 78%. Substantial differences in treatment planning outcome and dose distribution have previously been noted, indicating a need for not only VT ablation expertise but specifically expertise in STAR delivery for radiation oncologists for an accurate radiation delivery.^39^ Moreover, delivery of high-doses of radiation to complex moving targets remains a substantial challenge in-off itself.^40,41^ Therefore, expertise not only in radiation oncology but specifically STAR is critical if good outcomes are to be reached in this new therapeutic modality. This should include prior training, benchmarking, and protocol development for the execution of STAR.^42^

### Stereotactic arrhythmia radioablation in the present

Stereotactic arrhythmia radioablation today is performed in a highly selected population and most frequently in tertiary care centres and in research settings. Of note, most survey participants primarily considered treating/referring patients within clinical trials and an additional 13% asked for more data before treating patients with this modality. As STAR remains an investigational therapy, ideally treatments should be performed within clinical trials or prospective registries to allow for assessment of long-term safety and efficacy. The STOPSTORM consortium is an international research platform that has been established with the aim to standardize treatment and follow-up to allow data collection for research purposes and research collaboration.^43,44^

### Barriers and perceived problems with stereotactic arrhythmia radioablation

Prior publications suggest that although long-term data are lacking, STAR could represent a valuable tool in the antiarrhythmic armamentarium of electrophysiologists for the treatment of refractory VT in patients with cardiomyopathy. Despite a significant number of participants being open to performing STAR at this time, most of the participants expect STAR to remain a bail-out procedure. A majority of participants described a lack of reliable outcome data after STAR or indicated a lack of knowledge about the treatment. Interestingly, despite being an investigational treatment and procedure and a relative lack of data in the field (e.g. ideal radiation dose, fractionation of doses/number of treatment sessions, etc.), only a minority (5.5%) believed that STAR did not work.

An interesting finding of this survey was the patient characteristics regarded as prohibitive to perform STAR. Stereotactic arrhythmia radioablation, with its short treatment duration and recovery time, presumably could be a good bail-out therapy especially in patients with advanced heart failure. Despite this, 70% viewed a life expectancy < 6 months as a contraindication for it. This contrasts with a recent expert consensus on the clinical role of STAR. In this publication, the authors indicated a strong agreement amongst themselves that STAR should be considered in a compassionate use setting in patients with advanced heart failure and no alternative therapy options.^25^ The low acceptance of STAR for patients with poor prognosis could be possibly due to the relatively longer time to effect expected after treatment compared with an immediate effect of CA. Although radiation-induced fibrosis indeed develops only after 1–2 months, the antiarrhythmic effect is often seen within days to weeks after STAR delivery, leading to the assumption that additional antiarrhythmic mechanisms may play a role.^9,12,15^ Moreover, the commonly applied 20–25 Gy during STAR would not be sufficient to cause any transmural fibrosis.^5,7^

Despite the majority of participants viewing recurrent VT/VF as the primary treatment indication, the presence of an ICD was only a requirement for 39% of the participants. As mid-term VT/VF recurrence rates are high after STAR, ICD implantation in these patients should follow current guidelines.^23,27^ In addition, STAR will likely be reserved for patients with VT in structural heart disease and therefore application of STAR in patients without ICD will remain less likely.

### Stereotactic arrhythmia radioablation in the future

Despite its rapidly increasing and relatively widespread use, the future role of STAR is yet to be determined. The perceived future clinical role of STAR was very similar to its current role—mainly the treatment of VT/VF (with or without SHD) after failed CA or AAD, although treatment of atrial fibrillation has also been reported.^45^ This appears to be a relatively realistic view of a therapeutic solution that is driven by the limitation of the current therapies, i.e. penetration of deeply seated myocardial substrate. Since the majority of participants was convinced that STAR was effective (‘worked’), it is most likely the lack of RCTs and long-term safety data that limits its clinical application. A clinical guidelines/consensus document was viewed as the second most important publication after RCTs for the clinical decision-making of participants. Of note, there are several ongoing RCTs in the field including RADIATE-VT (NCT05765175), CARA-VT (NCT05047198), and STAR-VT (NCT04612140) and an EHRA consensus document will be published in 2024.

### Limitations

A selected group of participants were reached through the EHRA newsletter and social media. This is prone to selection bias as physicians that have exposure to this topic are more likely to respond and as participation in this voluntary survey. This is also reflected in the baseline characteristics of the respondents which were mostly electrophysiologists, followed by general cardiologists.

## Conclusions

Stereotactic arrhythmia radioablation is an investigational treatment option that is widely known and relatively accessible to European cardiologists today. At this point, it is most commonly applied in patients with recurrent VT/VF who have failed conventional therapies. Nevertheless, there are still barriers to its use, most importantly the lack of long-term outcome data and a lack of knowledge about the treatment; more must be understood about the basic mechanism of action so that this treatment can be optimized for in-human use within clinical trials. Ongoing RCTs and the planned EHRA consensus document will help to define and provide further insight in the future role of this treatment.

## Supplementary Material

euae110_Supplementary_Data

## Data Availability

The data that support the findings of this study are available on reasonable request from the corresponding author.
