# Supplementary material for: Stereotactic arrhythmia radioablation and its implications for modern cardiac electrophysiology: results of an EHRA survey
Source: Europace. 2024 Apr 26;26(5):euae110. doi: 10.1093/europace/euae110 (PMC11086561; doi:10.1093/europace/euae110)
Supplement: euae110_Supplementary_Data [file euae110_supplementary_data.pdf]

EHRA European Heart Rhythm Association

STereotactic Arrhythmia Radioablation and its place in modern cardiac ElectroPhysiology

Introduction

Dear colleague,
EHRA is conducting the STAR in EP survey on the role of Stereotactic Arrhythmia Radioablation (STAR) on your daily clinical decision making and your opinion about its role in the future of cardiology.
The aims of this survey are:
- to assess awareness of STAR among cardiologists and its subspecialties
- to inquire the access to STAR
- to understand what the current treatment and referral practices are for STAR
- to identify barriers to the application of STAR in clinical practice.
- to identify the needs of the cardiology community for future research and publications in STAR
- to discuss thoughts about the clinical potential of STAR

The survey will keep you occupied for about 10 minutes. Your answers will be considered anonymous.
If you have any comments or questions about the survey, feel free to contact us:
boldizsar.kovacs@usz.ch

Thank you in advance for your contribution!
Boldizsar Kovacs, H. Immo Lehmann on behalf of the EHRA Scientific Initiatives Committee.

This survey is about Stereotactic Arrhythmia Radioablation and its current and future role in cardiology. Several forms of radiotherapy exist. Most commonly, external radiation beams are used. STAR is a form of external radiotherapy with highly focused radiation beams called stereotactic body (or proton) radiotherapy (SBRT or SBPT) or stereotactic radiosurgery (SRS), which specifically targets myocardial tissue to treat arrhythmias. Synonyms used in literature are cardiac radioablation, cardiac SBRT, stereotactic arrhythmia radioablation, non-invasive cardiac radiation.

* 1. GDPR Disclaimer

We will not disclose your identity to any third party.

We comply with the European General Data Protection Regulation (GDPR) 2016/679. Any personal data processed in connection with this survey will be treated confidentially and only used by the ESC for the purposes of market research and not for promotion. Survey results will be kept for a maximum of 48 months for analysis and quality control purposes. We take all reasonable care to prevent any unauthorised access to your personal data. We respect your privacy and your right to access, modify, or remove your personal data. At any time, you can ask to know what personal data is being held. If you have any questions about data protection or require further information, please contact our data protection officer (DPO) at dpo@escardio.org.

You have the right to end your participation in this survey at any time.

Please confirm that you have read the above and agree to participate in this survey.

- ◯ yes
- ◯ no

EHRA European Heart Rhythm Association

STereotactic Arrhythmia Radioablation and its place in modern cardiac ElectroPhysiology

General Questions

* 2. Please indicate your age:

20 100

* 3. Please indicate your gender

- Male
- Female
- Non-binary
- Do not want to disclose

* 4. Which of the following is your primary speciality?

- General Cardiologist
- Electrophysiologist
- Heart Failure specialist
- Interventional Cardiologist
- Cardiac Imaging specialist
- Preventive or Sports Cardiology specialist
- Fellow in training
- Other (please specify)

5. How many years have you been in practice since you completed your cardiology training?

0 50

6. In which country do you practice?

* 7. Working place characteristics

- University Hospital
- Specialised Public Hospital
- District/Community Hospital
- Private Hospital
- Private Practice
- Other (please specify)

* 8. How familiar are you with Stereotactic Arrhythmia Radioablation (STAR)?

- ◯ I know about STAR and we perform it in our institution for clinical indications as bailout strategy
- ◯ I know about STAR and we perform it ONLY within approved research trials in our institution
- ◯ I know about STAR but we do not perform it in our institution
- ◯ This is the first time I hear/read about STAR

Your awareness of STAR & access to STAR

* 9. How many patients have you treated with or referred for STAR thus far?

0 100

* 10. What were the indications for STAR in these patients (multiple choice)

- [ ] Sustained ventricular tachyarrhythmia (i.e. ventricular tachycardia or VT/VF) in patients with structurally abnormal hearts
- [ ] VT/VF in patients WITHOUT structural heart disease
- [ ] Premature ventricular beats (PVC)
- [ ] Not applicable (I have not treated or referred any patients for STAR)
- [ ] Other (please specify)

11. Have you followed-up patients after treatment with STAR?

- ( ) Yes
- ( ) No

Current application of STAR

* 12. In your opinion, what is the role of STAR in the current management of ventricular arrhythmias?

- Purely experimental/investigational treatment
- Alternative to antiarrhythmic drugs or catheter ablation
- Bail-out treatment when other treatment options are contraindicated or not possible
- Adjunctive to other antiarrhythmic treatments in case of recurrent VT/VF
- Adjunctive to other antiarrhythmic treatments in case of recurrent VT/VF or PVC
- n/a (i.e. never heard of it)
- Other (please specify)

13. Based on my current knowledge and experience

	Strongly agree	Agree	Neutral	Disagree	Strongly disagree
I would consider performing STAR in my own institution for a either clinical indication (outside of a study protocol).	○	○	○	○	○
I would consider performing STAR in my own institution (inclusion in a study)	○	○	○	○	○
I would consider referring a patient for STAR (clinical indication)	○	○	○	○	○
I would consider referring a patient for STAR (inclusion in a study)	○	○	○	○	○

14. In your opinion, which are the reasons why STAR may not be adopted as an (experimental) treatment ? (multiple choice)

- Lack of knowledge about this treatment
- Lack of reliable outcomes data on STAR
- I do not believe this treatment works
- Concern of toxicity and side effects
- I do not know where the next centre is which offers STAR to patients
- Other (please specify)

* 15. Which further studies/publications would you like to see to facilitate your clinical decisions-making regarding STAR? (please put in order of priority - 1 most important, 5 least important)

- EHRA (or ESC) consensus document and expert recommendations on clinical use of STAR
- Prospective clinical outcome data demonstrating safety (>2 years follow-up)
- Randomized clinical trial in treatment naïve patients with VT/VF comparing STAR to ablation therapy and/or antiarrhythmic drug therapy
- Randomized clinical trial in patients with recurrent VT/VF after ablation therapy and/or antiarrhythmic therapy comparing STAR to repeat ablation therapy/escalation of antiarrhythmic drug therapy
- Other type of publication

* 16. Which inclusion criteria would you consider necessary for STAR ? (multiple choice)

- Structural heart diseases (ischemic cardiomyopathy or non-ischemic cardiomyopathy)
- Placement of an ICD
- Recurrent monomorphic VT (>3 episodes in the previous 3 months)
- Electrical strom
- Optimal antiarrhythmic medication
- One or more previous catheter ablation procedures or contraindication against ablation
- Other (please specify)

* 17. Which exclusion criteria would you consider prohibitive for STAR? (multiple choice)

- [ ] Polymorphic VT / ventricular fibrillation
- [ ] Temporary or genetic causes for VT
- [ ] Eligibility for catheter ablation
- [ ] Advanced heart failure (NYHA class IV)
- [ ] ICD malfunction
- [ ] Prior chest irradiation
- [ ] Life expectancy <6 months
- [ ] Pregnant or breastfeeding
- [ ] Other (please specify)

* 18. In your opinion, where should STAR be performed?

- ◯ Any hospital that offers this form of radiotherapy (stereotactic body radiotherapy) and has the radiation oncology expertise
- ◯ Any hospital that has the expertise in both VT ablation and expertise to perform stereotactic body radiotherapy
- ◯ Any hospital that has the expertise in both VT ablation and specifically STAR
- ◯ STAR should only be performed in an academic (i.e. research) setting
- ◯ STAR should not be performed anywhere

Future of STAR in cardiology and electrophysiology

19. How would you rank the possible advantages of STAR? (please put in order of priority - 1 most important, 6 least important)

- Higher efficacy than other available treatment options (ablation, antiarrhythmic drug therapy, etc.)
- Non-invasive treatment approach with overall low expected acute and short-term procedural risks
- Short treatment time (~20-40 minutes)
- Efficacy for arrhythmias that are not amenable to either catheter ablation or antiarrhythmic drugs
- Shorter expected recovery time from procedure allowing early (e.g. same day) discharge as compared to ablation
- Reduction of antiarrhythmic drug dosage

20. What in your opinion is the most appropriate way to determine the myocardial tissue that should be targeted with STAR for the treatment of ventricular arrhythmia (multiple choice)

- ☐ 3D electroanatomic data created using invasive mapping catheters
- ☐ Cross-sectional imaging (computed tomography, cardiac magnetic resonance imaging, etc.)
- ☐ Body surface mapping (or other similar forms of non-invasive VT/VF and PVC mapping tools)
- ☐ 12-lead ECG or Holter of VT/VF and PVC
- ☐ I don’t know
- ☐ Other (please specify)

* 21. Currently STAR is mainly considered a ‘last resort’ treatment for sustained VT/VF in structural heart disease. In your opinion, what will be the MOST LIKELY clinical role of STAR in the next 5-10 years in the following arrhythmias:

	First-line	Second-line	“Last resort/bail-out”t of ventricular tachyarrhythmias in structural heart disease	No role
VT/VF in all patients	◯	◯	◯	◯
VT/VF in patients with structurally abnormal hearts	◯	◯	◯	◯
PVC in all patients	◯	◯	◯	◯
PVC in patients with structurally abnormal hearts	◯	◯	◯	◯
Atrial arrhythmias (e.g. atrial fibrillation and flutter)	◯	◯	◯	◯

Thank you

Dear Colleague,
thank you for completing the STAR in EP survey.

Your input is very much appreciated and will help us to evaluate the current role and future of STAR in Cardiology and Electrophysiology. Please spread the word among your Cardiology and EP colleagues to answer this survey (https://www.surveymonkey.com/r/_S_T_A_R). It is not restricted to EHRA/ESC members or physicians practicing in Europe.

If you have any comments or questions on the survey, feel free to contact us:
boldizsar.kovacs@usz.ch
Thanks again for your contribution,

Boldizsar Kovacs, H. Immo Lehmann on behalf of the EHRA Scientific Initiatives Committee.
